# Differential Requirement for the CD45 Splicing Regulator hnRNPLL for Accumulation of NKT and Conventional T Cells

**DOI:** 10.1371/journal.pone.0026440

**Published:** 2011-11-04

**Authors:** Mehmet Yabas, Dale I. Godfrey, Christopher C. Goodnow, Gerard F. Hoyne

**Affiliations:** 1 Department of Immunology, The John Curtin School of Medical Research, The Australian National University, Canberra, Australia; 2 Department of Microbiology and Immunology, The University of Melbourne, Parkville, Australia; Centre de Recherche Public de la Santé (CRP-Santé), Luxembourg

## Abstract

Natural killer T (NKT) cells represent an important regulatory T cell subset that develops in the thymus and contains immature (NK1.1^lo^) and mature (NK1.1^hi^) cell subsets. Here we show in mice that an inherited mutation in heterogeneous ribonucleoprotein L-like protein (hnRNPLL^thunder^), that shortens the survival of conventional T cells, has no discernible effect on NKT cell development, homeostasis or effector function. Thus, *Hnrpll* deficiency effectively increases the NKT∶T cell ratio in the periphery. However, *Hnrpll* mutation disrupts CD45RA, RB and RC exon silencing of the *Ptprc* mRNA in both NKT and conventional T cells, and leads to a comparably dramatic shift to high molecular weight CD45 isoforms. In addition, *Hnrpll* mutation has a cell intrinsic effect on the expression of the developmentally regulated cell surface marker NK1.1 on NKT cells in the thymus and periphery but does not affect cell numbers. Therefore our results highlight both overlapping and divergent roles for hnRNPLL between conventional T cells and NKT cells. In both cell subsets it is required as a *trans*-acting factor to regulate alternative splicing of the *Ptprc* mRNA, but it is only required for survival of conventional T cells.

## Introduction

Natural Killer T (NKT) cells are a specialized subset of T lymphocytes that have the ability to regulate the immune response in a range of diseases [1], [2]. Unlike conventional T cells, NKT cells express an invariant T-cell receptor α chain (Vα14Jα18 in mice and Vα24Jα18 in humans) that is paired with a limited repertoire of Vβ chains (Vβ8.2, Vβ7, and Vβ2 in mice and Vβ11 in humans) [2], [3]. The antigen receptor of NKT cells recognizes glycolipid antigens, such as α-galactosylceramide (α-GalCer), presented by the nonclassical MHC-I like molecule CD1d [2], [4], [5]. NKT cells are positively selected from CD4^+^ CD8^+^ double positive (DP) thymocytes in a CD1d dependent manner in the thymus [6] and pass through four developmental stages that can be distinguished on the basis of CD24, CD44 and NK1.1 expression [2], [3]. The most immature cells are CD24^+^ but lack expression of CD44 and NK1.1 (stage 0) [7], and these give rise to CD24^−^ cells that are CD44^lo^ and NK1.1^lo^ (stage 1). These differentiate to become CD44^hi^ NK1.1^lo^ cells (stage 2) at which point they can be either exported to the periphery or continue to mature in the thymus [8], [9]. The final stage of maturation coincides with up-regulation of NK1.1 to become CD44^hi^ NK1.1^hi^ (stage 3) cells [8], [9]. The development and the maintenance of stable numbers of mature NKT cells in the thymus and periphery is determined by a wide range of factors including transcription factors (e.g. c-Myc, Egr2, PLZF, T-Bet) and cytokine signaling via IL-15 and TGF-β [2], [3]. The development and function of NKT cells can also be influenced by costimulatory molecules such as SLAM/SAP, CD80/CD86 and ICOS [2], [3]. Another important feature of NKT cells is that upon activation they are able to rapidly produce a diverse range of cytokines and this gives rise to an array of functionally distinct NKT cell subsets in both mice and humans that can be distinguished on the basis of the patterns of cytokine secretion [10], [11], [12]. Because NKT cell numbers are critical to the outcome of many diseases [1], and that the numbers vary widely between individuals [3], it is crucial that we understand how the development and function of NKT cells differs from that of other T cell subsets.

The heterogeneous nuclear ribonucleoprotein L-Like, hnRNPLL, is a member of hnRNP protein family that is essential for mRNA alternative splicing in T cells [13], [14], [15]. The role of hnRNPLL was revealed by a recessive loss of function mouse mutation isolated in an *N*-ethyl-*N*-nitrosourea (ENU) mutagenesis screen, *thunder* (*Hnrpll* ^thu/thu^) [15]. This mutation disrupts the first of three RNA-recognition motif domains in the hnRNPLL protein that binds to activation-responsive silencing (ARS) elements in the variably expressed exons 4, 5 and 6 of *Ptprc* [16], [17]. As a result, there is a failure to silence the inclusion of these exons in naïve and memory T cells so that cell surface CD45 protein shifts from the normal isoforms on T cells, CD45RB and RO, to forms such as CD45RA and CD45RC that are normally not found on T cells [15]. The *Hnrpll* ^thu^ mutation does not affect conventional αβ T cell differentiation in the thymus but it greatly shortens the survival of naïve and memory αβ T cells in the peripheral lymphoid tissues [15]. This is independent of the change CD45/*Ptprc* splicing, and must be explained by alternative splicing among the hundreds of other mRNAs that are regulated directly or indirectly by *Hnrpll* [15], [18]. NKT cells express the tyrosine phosphatase CD45, similar to conventional αβ T cells which can express up to eight different isoforms at the cell surface due to mRNA alternative splicing of three variable exons on the *Ptprc* mRNA [19]. The expression of the CD45 isoforms on conventional T cells is regulated in a development- and activation-dependent manner that is regulated by exon silencing by *Hnrpll* [13], [14], [15]. Here we examine the requirement for *Hnrpll* in NKT cells and show that although hnRNPLL is required for the splicing of CD45 isoforms in both αβ T cells and NKT cell lineages it does not affect NKT cell accumulation and survival, although it appears to play a role in maintaining basal expression of the differentiation marker NK1.1. Our studies highlight a fundamental difference between the action of hnRNPLL in conventional T cells and NKT cells.

## Results

### hnRNPLL is required for the splicing of CD45 isoforms in NKT cells

**Figure 1:**
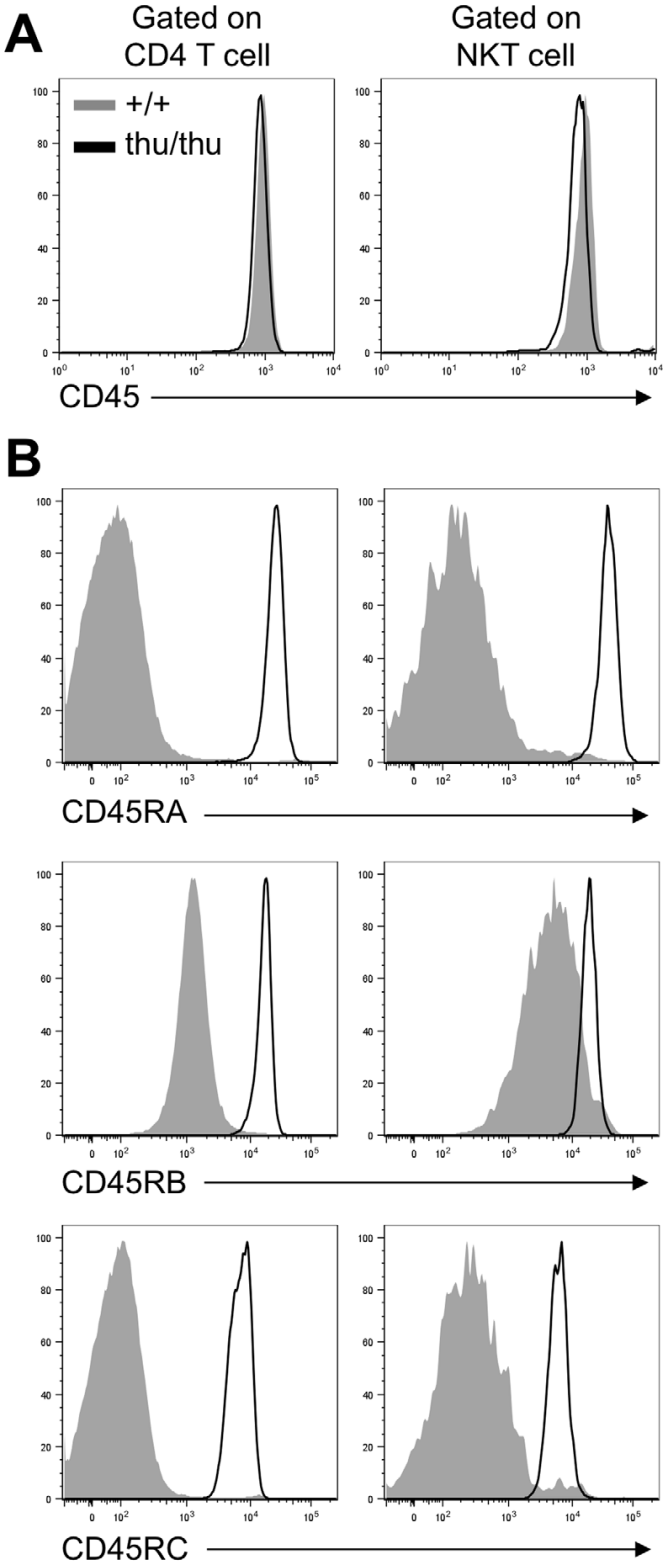
hnRNPLL is required for the splicing of CD45 isoforms in NKT cells. Representative overlay histograms of (A) CD45 and (B) CD45RA, CD45RB and CD45RC expressions on wild type (+/+) (shaded grey) and *Hnrpll* ^thu/thu^ (thu/thu) (black line) CD4 T cells and NKT cells in the thymus (Data are representative of two independent experiments with n = 2–3 mice per group in each).

Flow cytometric analysis of CD45 isoform expression on NKT cells was performed by staining thymus and spleen cells from wild type and *Hnrpll* ^thu/thu^ mice with α -GalCer-loaded CD1d tetramers and antibodies to TCRβ [20], [21]. This revealed that that there was high expression of CD45 and no difference in total CD45 staining intensity at the cell surface in *Hnrpll*-mutant NKT cells (Figure 1A). NKT cells from wild type mice expressed very little CD45RA or RC isoforms, which include the variable segments encoded in *Ptprc* exons 4 and 6 respectively, and intermediate levels of the CD45RB isoform derived from exon 5. This pattern of CD45 isoform expression is similar to that observed on conventional αβ T cells (CD4 cells in Figure 1B). By contrast, CD45RA, CD45RB and CD45RC isoforms were increased to between 10 and 100 times higher levels on NKT cells from *Hnrpll* ^thu/thu^ mice (Figure 1B). This result demonstrates that hnRNPLL is critical for silencing *Ptprc* exons 4, 5 and 6 within NKT cells, and *Hnrpll* mutation has a comparable effect on this process in NKT and conventional T cells.

### Normal NKT cell numbers in the thymus and periphery of *Hnrpll* ^thu/thu^ mice

**Figure 2:**
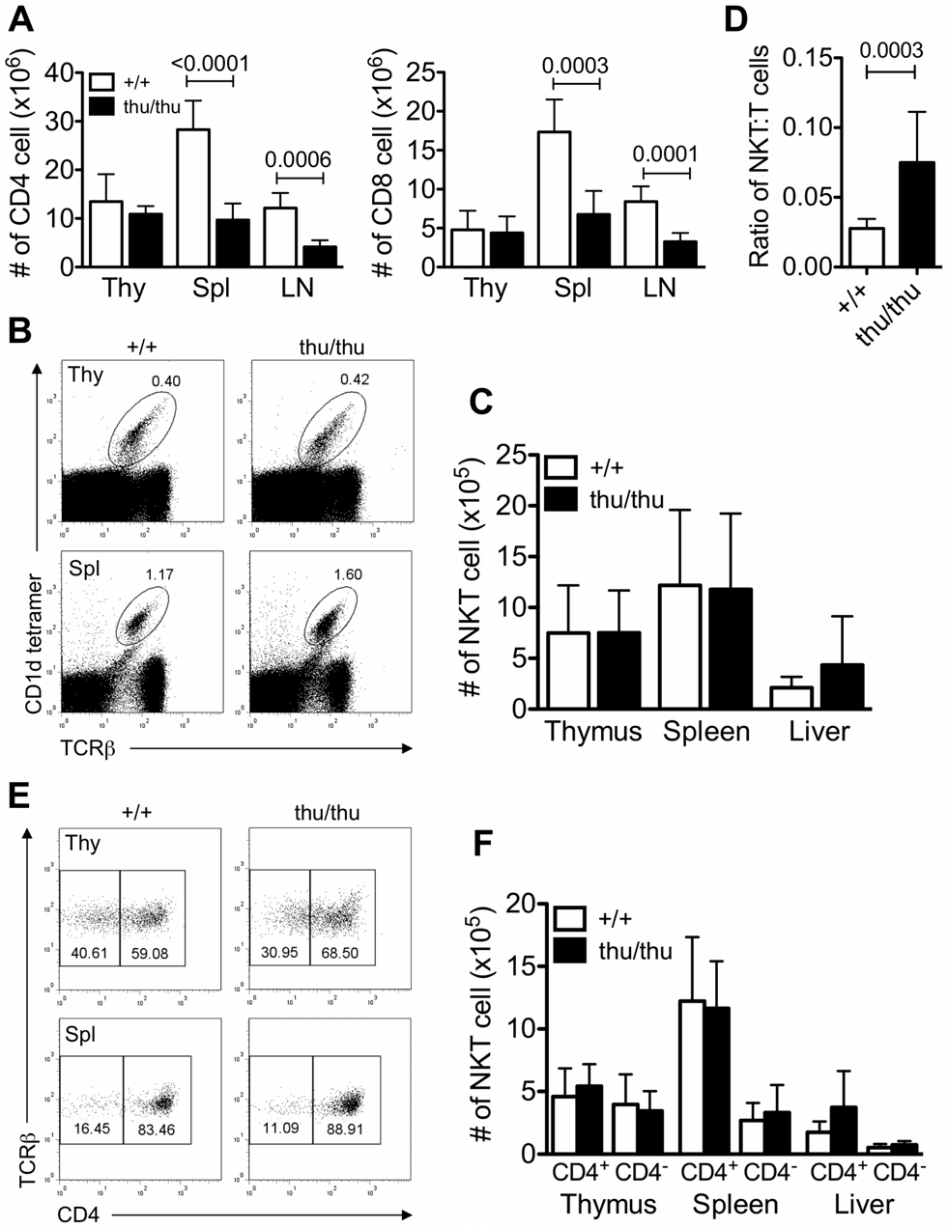
Normal numbers of NKT cell in the thymus and periphery of *Hnrpll* ^thu/thu^ mice despite of reduced number of conventional T cells in the periphery. (A) Graphs show absolute number of CD4 and CD8 conventional T cells in wild type (+/+) and *Hnrpll* ^thu/thu^ (thu/thu) mice (n = 9 for wild type and n = 8 for *Hnrpll* ^thu/thu^ analysed in two independent experiments). Bars represent the mean ± s.d. values. (B) Representative flow cytometric dot plots of NKT cells (α-GalCer-loaded CD1d tetramer^+^ TCRβ^+^) in the thymus (Thy) and spleen (Spl) in wild type (+/+) and *Hnrpll* ^thu/thu^ (thu/thu) mice. (C) Graph shows absolute number of NKT cells in the different tissues of wild type (+/+) and *Hnrpll* ^thu/thu^ (thu/thu) mice (n = 12 for wild type and n = 11 for *Hnrpll* ^thu/thu^ analysed in three independent experiments). Bars represent the mean ± s.d. values. (D) Graph shows the ratio of absolute number of NKT cells to absolute number of TCRβ^+^ conventional T cells (n = 12 for wild type (+/+) and n = 11 for *Hnrpll* ^thu/thu^ (thu/thu) analysed in three independent experiments). Bars represent the mean ± s.d. values. (E) Representative flow cytometric dot plots comparing the expression of CD4 versus TCRβ on CD1d-tetramer^+^ TCRβ^+^ NKT cells in the thymus, and spleen of wild type (+/+) and *Hnrpll* ^thu/thu^ (thu/thu) mice. (F) Graph shows absolute number of CD4^+^ and CD4^−^ NKT cell subsets in the thymus, spleen and liver of individual wild type (+/+) and *Hnrpll* ^thu/thu^ (thu/thu) mice (n = 9 for wild type and n = 8 for *Hnrpll* ^thu/thu^ analysed in two independent experiments). Bars represent the mean ± s.d. values.

Given that NKT cells and conventional T cells develop in the thymus from the same precursor, we wanted to examine if the *Hnrpll* mutation affected NKT cell development, function and homeostasis. We stained the thymus, spleen and liver of wild type and *Hnrpll* ^thu/thu^ mice for conventional and NKT cells. Conventional T cells in the thymus were not significantly different in frequency or absolute number between wild type and *Hnrpll* ^thu/thu^ mice, but the mutation reduced the number of peripheral CD4^+^ and CD8^+^ T cells (Figure 2A) which we have previously shown is caused by loss of naive CD4^+^ and CD8^+^ cells [15]. By comparison, *Hnrpll* mutation did not affect the number of NKT cells in the thymus, spleen or liver (Figure 2B&C). However, we observed that NKT cells from *Hnrpll* ^thu/thu^ mice have a reproducibly lower level of TCRβ expression as measured by intensity of tetramer staining compared to wild type cells. Thus, in the spleen the ratio of absolute number of NKT cells to absolute number of TCRβ^+^ conventional T cells was significantly increased in *Hnrpll* ^thu/thu^ mice suggesting an important role for hnRNPLL in maintaining a normal NKT∶T cell ratio in the periphery (Figure 2D). Two subsets of NKT cells can be distinguished based on CD4 expression, namely CD4^+^ and CD4^−^ [3]. There was no significant difference in the number of CD4^+^ or CD4^−^ NKT cell subsets between wild type and *Hnrpll* ^thu/thu^ mice in the thymus, spleen or liver (Figure 2E&F). Thus, while NKT and conventional T cells share a requirement of hnRNPLL to control their CD45 isoforms, they differ in their hnRNPLL requirement for accumulating in normal numbers.

### Decreased NK1.1 during NKT cell development in *Hnrpll* ^thu/thu^ mice

**Figure 3:**
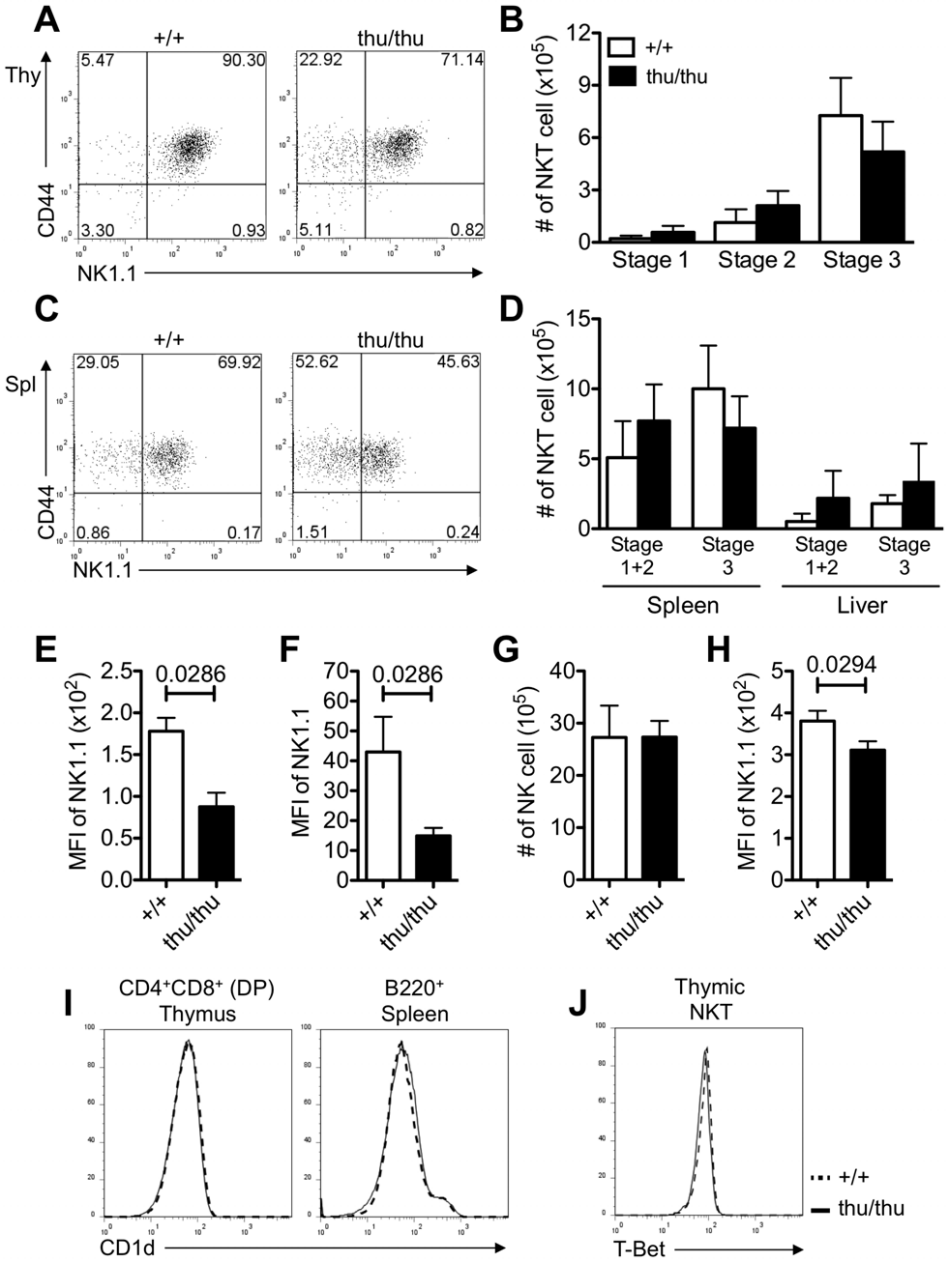
Decreased NK1.1 during NKT cell development in *Hnrpll* ^thu/thu^ mice. (A, C) Representative flow cytometric dot plots showing the development stages of NKT cell in the (a) thymus and (c) spleen in intact wild type (+/+) and *Hnrpll* ^thu/thu^ (thu/thu) mice based on expression of CD44 and NK1.1 that were gated on α-Galcer-Cd1d tetramer^+^ TCRβ^+^ NKT cells. (B, D) Graphs show the absolute number of the three development stages of NKT cell in the (b) thymus and (d) spleen and liver respectively (n = 9 for wild type and n = 8 for *Hnrpll* ^thu/thu^ analysed in two independent experiments). Bars represent the mean ± s.d. values. (E, F) Graphs show the geometric mean florescence intensity (MFI) of NK1.1 in the thymic and splenic NKT cells respectively (Data are representative of three independent experiments with n = 3–5 mice per group in each). Bars represent the mean ± s.d. values. (G) Graph shows the absolute number of NK1.1^+^ TCRβ^−^ NK cell in the spleen (Data are representative of three independent experiments with n = 3–5 mice per group in each). Bars represent the mean ± s.d. values. (H) Graph shows the geometric mean florescence intensity (MFI) of NK1.1 in the splenic NK cells (Data are representative of three independent experiments with n = 3–5 mice per group in each). Bars represent the mean ± s.d. values. (I) Representative overlay histograms comparing the expression of CD1d on DP thymocytes and B220^+^ cells in the spleen. (J) Representative overlay histograms showing the intracellular expression of T-Bet in NKT cells from the thymus of a wild type (dotted line) and *Hnrpll* ^thu/thu^ mouse (solid line) (Data are representative of two independent experiments with n = 3–5 mice per group in each).

NKT cells differentiate in the thymus and progress through well-defined stages that can be distinguished on the basis of expression of CD44 and NK1.1 [3]. We examined the developmental stages of NKT differentiation in the thymus and peripheral tissues of wild type and *Hnrpll* ^thu/thu^ mice and observed a slight increase in the mean frequency and absolute number of CD44^lo^ NK1.1^lo^ (Stage 1) and CD44^hi^ NK1.1^lo^ (Stage 2) NKT cells and a reduction in the mean frequency and number of mature CD44^hi^ NK1.1^hi^ (Stage 3) NKT cells in the thymus of *Hnrpll* ^thu/thu^ mice compared to wild type controls (Figure 3A&B). Stage 2 NKT cells can undergo maturation to stage 3 cells either in thymus or peripheral tissues. We also observed a similar trend with an increase in NK1.1^−^ NKT cells in the spleen of *Hnrpll* ^thu/thu^ mice with a corresponding reduction of NK1.1^+^ NKT cells compared to wild type animals (Figure 3C&D). However the change in cell numbers within the different cell subsets was not statistically significant, and rather than representing a defect in NKT cell maturation, they may simply be secondary to the fact that *Hnrpll* ^thu/thu^ NKT cells in the thymus and spleen had significantly lower NK1.1 expression (Figure 3E&F). NK cells in *Hnrpll* ^thu/thu^ mice also displayed a lower cell surface NK1.1 expression but, similar to the NKT cell population, no difference in the accumulation of these cells in the peripheral lymphoid tissues compared to conventional αβ T cells was detected (Figure 3G&H).

The positive selection of NKT cells occurs in a CD1d-dependent manner from DP thymocytes [6]. Analysis of CD1d expression in the thymus showed that the *Hnrpll* mutation does not affect the expression of CD1d on DP cells (Figure 3I). T-Bet (*Tbx21*) is another important factor in the transition from Stage 2 to Stage 3 cells by up-regulating CD122 expression [22]. Intracellular flow cytometric staining of thymic NKT cells revealed normal T-Bet expression in *Hnrpll* ^thu/thu^ NKT cells compared to wild type cells (Figure 3J). These results support the notion that hnRNPLL is not required for the development of NKT lineage in the thymus or periphery but is required for full induction of NK1.1.

### *Hnrpll* mutant NKT cells exhibit a cell autonomous decrease in NK1.1 in mixed bone marrow chimeras

**Figure 4:**
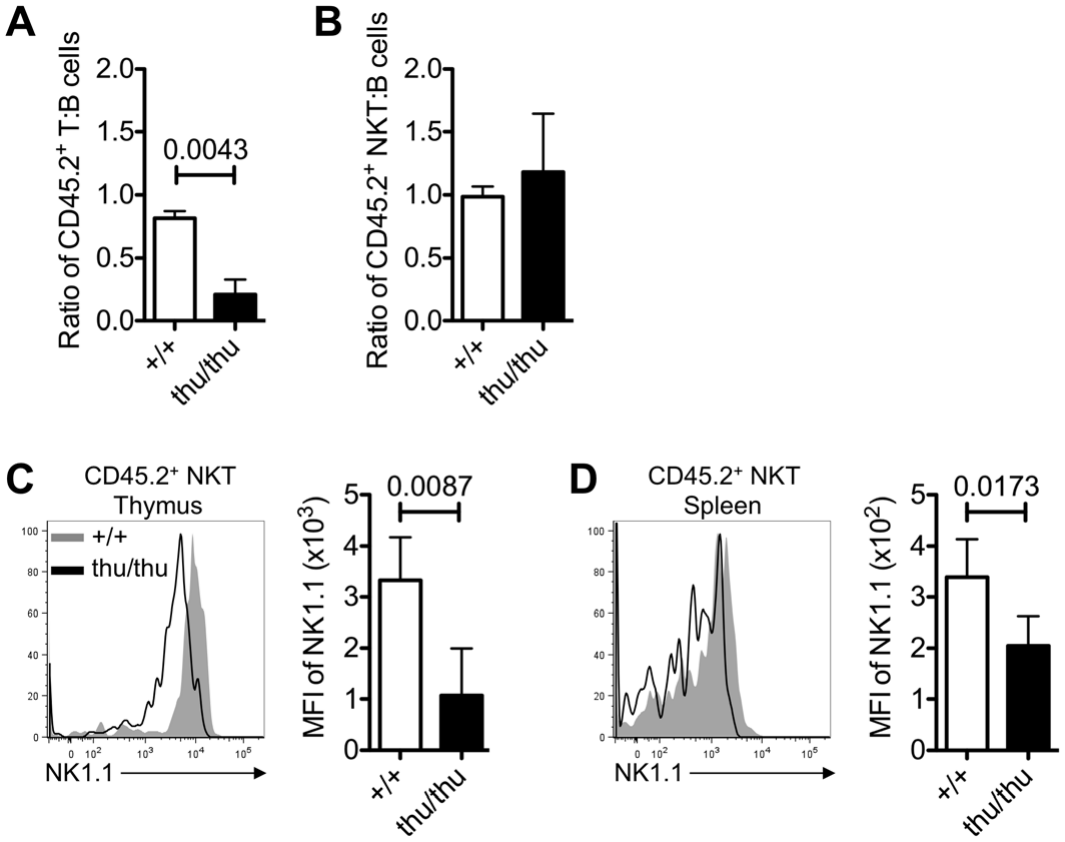
Normal contribution of *Hnrpll* ^thu/thu^ NKT cells, but a cell autonomous decrease in NK1.1 in the mixed bone marrow chimeras. Irradiated C57BL/6 mice were reconstituted with an equal mixture of CD45.1-marked wild-type bone marrow and CD45.2-marked *Hnrpll* ^thu/thu^ bone marrow cells. After hemopoietic reconstitution, recipient mice were analysed for B, NKT and conventional T cell populations. (A) Graph shows the ratio of CD45.2^+^ wild type and *Hnrpll* ^thu/thu^ derived T cells to CD45.2^+^ wild type and *Hnrpll* ^thu/thu^ derived B cells in the spleen of chimeric recipient mice. (B) Graph shows the ratio of CD45.2^+^ wild type and *Hnrpll* ^thu/thu^ derived NKT cells to CD45.2^+^ wild type and *Hnrpll* ^thu/thu^ derived B cells in the spleen of chimeric recipient mice. (C, D) Representative overlay histograms comparing the expression of NK1.1 on wild type (shaded grey) and *Hnrpll* ^thu/thu^ (black line) derived NKT cells in the (b) thymus and (c) spleen of individual recipient mice from mixed bone marrow chimeras. Graphs show the geometric mean florescence intensity (MFI) of NK1.1 in the CD45.2^+^ wild type or *Hnrpll* ^thu/thu^ derived NKT cells in the (b) thymus and (c) spleen of individual recipient mice from mixed bone marrow chimeras. (n = 6 for wild type and n = 5 for *Hnrpll* ^thu/thu^). Bars represent the mean ± s.d. values.

To investigate the possibility that homeostatic effects were masking a role for hnRNPLL in NKT cell development, mixed bone marrow chimeras (BMC) were used to investigate the importance of hnRNPLL on NKT and T cell development in a competitive situation. Irradiated C57BL/6 mice were reconstituted with an equal mixture of CD45.1-marked wild-type *Hnrpll* ^+/+^ C57BL/6 bone marrow and CD45.2 *Hnrpll* ^thu/thu^ C57BL/6 bone marrow cells. Recipient mice were analysed 12–14 weeks after injection by examining donor derived B cells, conventional T and NKT cells. The relative reconstitution of each chimeric mouse by *Hnrpll* ^thu/thu^ CD45.2^+^ hemopoietic stem cells was gauged by the percentage of B cells that were CD45.2^+^, since this lineage expresses very little *Hnrpll* and is not affected by the *Hnrpll* mutation [15]. The results showed that comparing the ratio of T cells to B cells in the recipient mice, there was a significant decrease in the proportion of conventional T cells in the spleen compared to wild type animals (Figure 4A). The competitive disadvantage of *Hnrpll* ^thu/thu^ derived conventional TCRαβ^+^ cells in the periphery is consistent with previous studies in mixed chimeras [15]. In contrast there was no significant difference in the ratio of wild type or *Hnrpll* ^thu/thu^ derived NKT cells to B cells in the spleen of recipient mice (Figure 4B), establishing that the loss of function of hnRNPLL did not affect the differentiation or long-term survival of mature NKT cells. Detailed analysis of *Hnrpll* ^thu/thu^ versus wild-type NKT cells in the mixed chimeras revealed that, consistent with the results shown above, the mean fluorescence intensity of NK1.1 expression was significantly lower on *Hnrpll* ^thu/thu^ derived NKT cells in the thymus (Figure 4C) and spleen (Figure 4D). Taken together, these data show that hnRNPLL is not required for the development or persistence of NKT cells in the immune system, in contrast to its role in conventional T cells, but it functions cell intrinsically to regulate NK1.1 expression in NKT cells.

### Mature NKT cells in *Hnrpll* ^thu/thu^ mice can survive normally in the presence of IL-15

**Figure 5:**
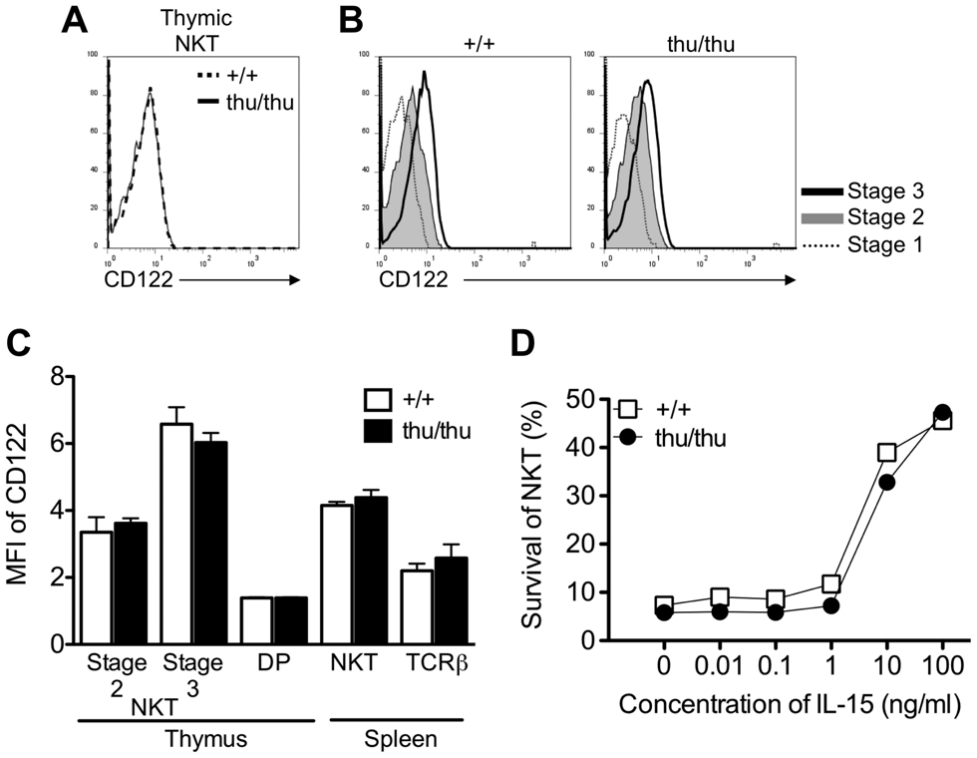
The *Hnrpll* mutation does not affect the up-regulation of CD122 in the developmental stages of NKT cell. (A) Representative overlay histograms showing the expression of CD122 on the surface of NKT cells from the thymus of a wild type and *Hnrpll* ^thu/thu^ mouse. (B) Representative overlay histograms comparing CD122 expression on stage 1 (CD44^lo^ NK1.1^lo^), stage 2 (CD44^hi^ NK1.1^lo^) and stage 3 (CD44^hi^ NK1.1^hi^) NKT cells in the thymus in wild type (left) and *Hnrpll* ^thu/thu^ mice (right). (C) Graph shows the geometric mean fluorescence intensity (MFI) of CD122 expression on stage 2 and 3 NKT cells and DP thymocytes in the thymus and NKT cells and TCRβ^+^ cells in the spleen. Bars represent the mean value of each group ± s.d. from one of two independent experiments with a group of n = 3–5 mice per group in each. (D) *In vitro* survival of NKT cells culture in the presence of varying concentrations of IL-15. Data shows the percentage of viable cells by 7-AAD exclusion after 3 days of culture for wild type (+/+) and *Hnrpll* ^thu/thu^ (thu/thu) thymic NKT cells. Graph from one of three independent experiments represents the average of viable cells recovered from duplicate cultures with 2 or 3 mice per group.

The maturation of NKT cells from the NK1.1^lo^ to NK1.1^hi^ stage coincides with expression of the CD122 that confers responsiveness to IL-15 and leads to increased expression of NK1.1 and their long term survival *in vivo* [23], [24]. Based on our hypothesis that hnRNPLL is required to regulate NK1.1 expression but does not influence NKT cell maturation, a prediction is that the *Hnrpll* ^thu/thu^ NKT cells should show comparable expression of CD122 and survival in the presence of IL-15 compared to wild type cells. Indeed, the *Hnrpll* mutation did not affect the expression of CD122 as determined by flow cytometric staining of NKT cells between wild type and *Hnrpll* ^thu/thu^ mutant cells (Figure 5A–C). Next we examined the survival of wild type and *Hnrpll* ^thu/thu^ thymic NKT cells in the presence or absence of IL-15 for 3 days. The viability of the cells was analysed by staining with 7-AAD and quantitated by flow cytometry. We found no difference in the survival between wild type or *Hnrpll* ^thu/thu^ NKT cells to IL-15 *in vitro* at each concentration of IL-15 tested (Figure 5D).

### *Hnrpll* mutation does not disrupt cytokine by NKT cells

**Figure 6:**
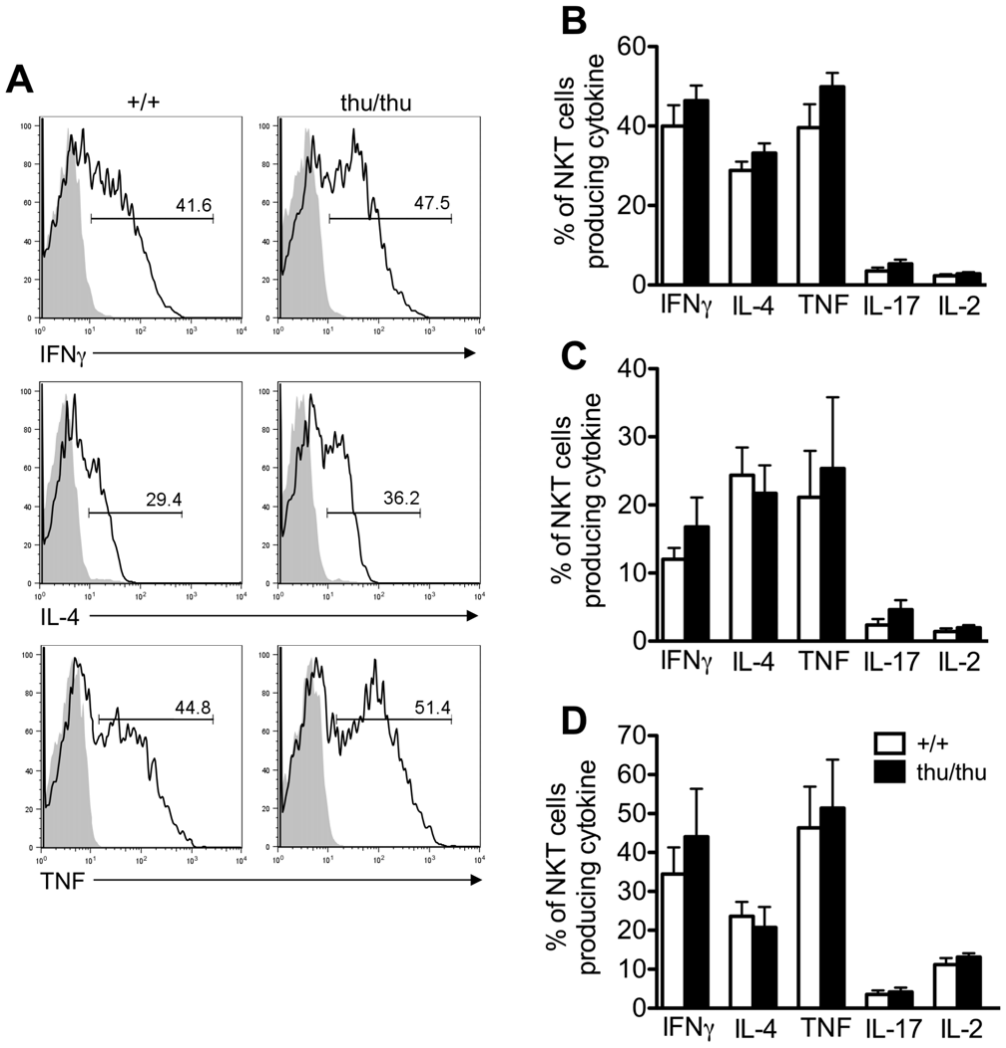
The *Hnrpll* mutation does not disrupt cytokine production by thymic, splenic and hepatic NKT cells. (A) Representative histograms showing intracellular cytokine production by α-GalCer-CD1d tetramer^+^ TCRβ^+^ NKT cells from wild type (+/+) and *Hnrpll* ^thu/thu^ (thu/thu) mice that were either resting (shaded grey) or following activation with PMA and ionomycin (black line). (B) Frequency of cytokine producing cells of total NKT cells in the thymus of wild type and *Hnrpll* ^thu/thu^ mice. (C) Frequency of cytokine producing cells of total NKT cells in the spleen of wild type and *Hnrpll* ^thu/thu^ mice. (D) Frequency of cytokine producing cells of total NKT cells in the liver of wild type and *Hnrpll* ^thu/thu^ mice. Bars represent the mean value of each group ± s.d. from one of two independent experiments with a group of n = 3–5 mice per group in each.

Immature and mature NKT cells are able to rapidly secrete cytokines following activation [2], [25]. We examined the cytokine production by wild type and *Hnrpll* ^thu/thu^ NKT cells following short-term activation of freshly isolated cells in the thymus, spleen and liver. NKT cells were stimulated *in vitro* in the presence of PMA and ionomycin for 3–4 h and intracellular cytokine analysis was examined for IFN-γ, IL-4, TNF, IL-17 and IL-2 (Figure 6). There was little or no difference in the patterns of IFN-γ, IL-4, TNF, IL-17 and IL-2 secretion between the *Hnrpll* ^thu/thu^ and wild type thymic NKT cells (Figure 6A&B). In addition wild type and *Hnrpll* ^thu/thu^ derived NKT cells secreted comparable levels of IFN-γ, IL-4, TNF, IL-17 and IL-2 in the spleen and liver (Figure 6C&D). Overall the results indicate that the *Hnrpll* mutation does not affect cytokine production by *in vitro* activated NKT cells.

## Discussion

hnRNPLL has previously been shown to control the survival of naive CD4^+^ and CD8^+^ conventional T cells in the peripheral circulation [15]. In this report we show that hnRNPLL is required for exon silencing of the variable exons of the *Ptprc* nascent mRNA in NKT cells and this function is conserved across multiple cell lineages in the immune system [15]. In contrast to conventional CD4^+^ and CD8^+^ TCR αβ^+^ cells we did not observe any effect of the hnRNPLL^thu/thu^ allele on NKT cell development, effector cell responses or the maintenance of stable NKT cell numbers in the lymphoid and non-lymphoid tissues. However, *Hnrpll* ^thu/thu^ derived NKT and NK cells displayed lower expression of the developmental marker NK1.1 that appears to be independent of IL-15 and TCR signaling. This study reveals a divergent role for hnRNPLL within different T cell lineages, which presumably reflects a difference in their dependence on alternative mRNA splicing.

The NK1.1^lo^ to NK1.1^hi^ transition represents an important developmental checkpoint in NKT cell maturation that relies on both TCR and IL-15 signaling [23], [24], [26], [27], but the homeostatic proliferation and maintenance of mature NK1.1^+^ cells in the immune system is independent of TCR-CD1d signaling and relies instead on the availability of IL-15 and signaling through the IL-15R on NKT cells [24]. Importantly NKT cells are not the only cells in the immune system that rely on IL-15 for their homeostasis, memory CD8^+^ CD44^hi^ T cells and NK cells proliferate strongly in response to IL-15 [11], [28].

CD45 is an abundant tyrosine phosphatase expressed by leukocytes and undergoes alternative splicing of the three variable exons 4, 5 and 6 to yield multiple isoforms. The two related proteins hnRNPL and hnRNPLL function as *trans* acting factors that regulate exon silencing of the variable exons of *Ptprc* mRNA in CD4^+^ and CD8^+^ T cells [13], [14], [15], [17], [29]. As shown here wild type NKT cells normally express only low levels of the high molecular weight CD45 isoforms CD45RA and CD45RC at the cell surface as they rely on a functional hnRNPLL to mediate the exon silencing of *Ptprc* mRNA of the three variable exons. The *Hnrpll* mutation disrupts binding to ARS sequences found in *Ptprc* exons 4–6, leads to inclusion of these exons and a dramatic shift to CD45RA^+^, RB^+^ and RC^+^ isoforms in NKT cells. Despite the inappropriate expression of the high molecular CD45 isoforms, this did not affect NKT cell development or effector responses following activation. This is similar to our recent observation that constitutive expression of CD45 isoforms in conventional T cells of hnRNPLL mutant mice was also not responsible for the disruption to T cell homeostasis observed in these mice [18].

The mechanism responsible for the lower NK1.1 expression in *Hnrpll* ^thu/thu^ NKT cells is unclear, but our data would suggest that it is independent of T-Bet and CD122 as the expression of both proteins was comparable between wild type and mutant cells. A focus of future studies would be to identify the specific *cis-* or *trans* acting factor(s) that directs NK1.1 gene expression, to identify if they are targets of mRNA splicing controlled by hnRNPLL. The biological significance of NK1.1 expression itself by NKT cells is unclear. A population of mature NK1.1^−^ NKT cells exists in the periphery of normal mice and these cells are capable of producing similar amounts of cytokines to their NK1.1^+^ counterparts [30]. However, antibody mediated NK1.1 cross-linking is capable of activating NKT cells to produce IFN-γ [31] and NK1.1 expression levels transiently decrease following TCR mediated NKT cell activation [32], [33]. The human counterpart of this molecule (CD161c) is also a known maturation marker for human NKT cells [3], but again, the functional significance of this molecule in humans is unknown. Thus, whether the NK1.1 cell surface receptor is important for the function of NKT cells remains an interesting question that needs to be resolved.

In conclusion, our studies have demonstrated that hnRNPLL is necessary for CD45 alternative splicing in both conventional T and NKT cells. The studies presented here also reinforce that we still have a lot to learn about mRNA splicing in the immune system. In naive conventional T cells it controls their long-term survival and homeostasis, but in NKT cells it does not affect lineage development or effector cell differentiation but has an effect on regulation of an important developmental marker NK1.1.

## Materials and Methods

### Mice

The *Hnrpll* ^thu/thu^ mouse strain has been previously described and was generated and maintained on a C57BL/6 background [15]. The C57BL/6 (B6) inbred mouse strain was originally obtained from Stanford University. All mice were housed and maintained in specific pathogen free conditions at the Australian Phenomics Facility and all animal procedures were approved by the Australian National University Animal Ethics and Experimentation Committee. The following is the ethics number approval for the proposal at the time: Proposal Nos: J.IG.31.04.

### Cell Preparation and Flow Cytometry

Single cell suspensions from thymus, spleen and liver were labelled with mixtures made of the following antibodies: PE labelled CD1d tetramer loaded with α-galactosylceramide (Sapphire Bioscience) was produced in house using a construct provided by M. Kronenberg (La Jolla Institute for Allergy and Immunology, San Diego, CA), FITC labelled anti-TCRβ (clone H57-597) from BD Pharmingen or APC-Cy7 labelled anti-TCRβ (clone H57-597) from Biolegend, APC, FITC or PE-Cy7 labelled anti-NK1.1 (clone PK136) from BD Pharmingen, Alexa Flour 700 labelled anti-CD4 (clone RM4-5) or APC-Cy7 labelled anti-CD4 (clone GK1.5) from BD Pharmingen, PE-Cy7 labelled anti-CD8 (clone 53-6.7) from Biolegend, Pacific Blue labelled anti-CD44 (clone IM7) from Biolegend, Alexa Flour 700 labelled anti-CD45.1 (clone A20) from Biolegend, PerCP-Cy5.5 labelled anti-CD45.2 (clone 104) from BD Pharmingen, FITC labelled anti-CD45 (clone 30-F11) from BD Pharmingen, PerCP-Cy5.5 labelled anti-mouse/human T-Bet (clone eBio4B10) from eBioscience, FITC labelled rat anti-mouse CD122 from BD Pharmingen, PerCP labelled anti-CD45R/B220 (clone RA3-6B2) from BD Pharmingen, Biotin anti-CD45RA (clone 14.8) from BD Pharmingen, FITC labelled anti-CD45RB (clone 16A) from BD Pharmingen, PE labelled anti-CD45RC (clone DNL-1.9) from BD Pharmingen, Qdot 605 streptavidin conjugate from Invitrogen, PE labelled anti-CD1d (clone 1B1) from BD Pharmingen.

Cell suspensions were incubated with appropriate mixture of antibodies at 4°C at the dark for 30 mins. Cells then washed with flow cytometry buffer containing 2% Bovine Serum and 0.1% NaN_3_ in PBS. Flow cytometry was performed on a LSR II (BD Biosciences) and analysed using FlowJo (Tree Star) software.

### Generation of Bone Marrow Chimeras

Bone marrow chimeric mice were generated by the injection of C57BL/6 (CD45.1 or CD45.2) and *Hnrpll* ^thu/thu^ (CD45.2) donor bone marrow cells into irradiated C57BL/6 (CD45.1) recipients. The irradiation dose was 2×450 rad and mixed at a 1∶1 ratio of donor cells injected intravenously at a 200 µL/mouse into the tail veins of recipients. Mice were sacrificed 12–14 weeks after the transplantation for ex vivo analysis of the immune system.

### Survival assay

Thymic NKT cells were cultured *in vitro* in the presence or absence of IL-15 (R&D Systems) for 3 days at 37°C and 5% CO_2_. The cells were harvested from the plate and stained with antibodies to cell surface markers and 7-aminoactiomycin (7-AAD) (Invitrogen) and the cell viability was analysed by flow cytometry as described above.

### Cell culture and intracellular cytokine staining

For *in vitro* stimulation assay, single cell suspensions were made from thymus, spleen and liver and cells were cultured in RPMI 1640 medium (Gibco) supplemented with penicillin, streptomycin, Glutamax, 1 mM sodium pyruvate (Sigma Aldrich), 0.1 mM nonessential amino acids (Sigma Aldrich), 10 mM Hepes (Sigma Aldrich), 55 µM 2-ME (Gibco) and 10% FCS in a 24-well plate in the presence or absence of 50 ng/ml PMA (Sigma), 500 ng/ml ionomycin (Sigma), and Golgi Stop (BD Pharmingen) for 3–4 h at 37°C and 5% CO_2_. After culture, surface markers were stained as detailed above prior to fixation of the cells. Cells were washed and stained intracellularly with FITC labelled anti-IL-4 (clone 11B11) from BD Pharmingen, Alexa Flour 700 labelled anti-IL-2 (clone JES6-5H4) from Biolegend, APC labelled anti-TNF (clone MP6-XT22) from eBioscience, Alexa Flour 700 labelled anti-IL-17A (clone TC11-18H10.1) from Biolegend and APC labelled anti-IFN-γ (clone XMG1.2) using the eBioscence Fixation/Permeabilization kit according to the manufacturer's instructions. Flow cytometry analysis of samples was performed on a LSR II (BD Biosciences) and analysed using FlowJo (Tree Star) software.

### Statistical analyses

Statistical analysis was performed with the Mann-Whitney Rank-Sum U-test using GraphPad Prism software.
